# Comprehensive interpretation of in vitro micronucleus test results for 292 chemicals: from hazard identification to risk assessment application

**DOI:** 10.1007/s00204-022-03286-2

**Published:** 2022-04-21

**Authors:** Byron Kuo, Marc A. Beal, John W. Wills, Paul A. White, Francesco Marchetti, Andy Nong, Tara S. Barton-Maclaren, Keith Houck, Carole L. Yauk

**Affiliations:** 1 Environmental Health Science and Research Bureau, Environmental and Radiation Health Sciences Directorate, Healthy Environment and Consumer Safety Branch, Health Canada, Ottawa, ON Canada; 2 Bureau of Chemical Safety, Food Directorate, Health Products and Food Branch, Health Canada, Ottawa, ON Canada; 3 Biominerals Research, Department of Veterinary Medicine, University of Cambridge, Cambridge, CB3 0ES UK; 4 Existing Substances Risk Assessment Bureau, Safe Environments Directorate, Healthy Environment and Consumer Safety Branch, Health Canada, Ottawa, ON Canada; 5 National Center for Computational Toxicology, Office of Research and Development, U.S. Environmental Protection Agency, Research Triangle Park, NC 27711 USA; 6 Present Address: Department of Biology, University of Ottawa, 30 Marie Curie Private, Room 269, Ottawa, ON K1N 6N5 Canada

**Keywords:** Micronucleus, Genotoxicity, Benchmark dose, Toxicokinetics, IVIVE, Point of departure

## Abstract

Risk assessments are increasingly reliant on information from in vitro assays. The in vitro micronucleus test (MNvit) is a genotoxicity test that detects chromosomal abnormalities, including chromosome breakage (clastogenicity) and/or whole chromosome loss (aneugenicity). In this study, MNvit datasets for 292 chemicals, generated by the US EPA’s ToxCast program, were evaluated using a decision tree-based pipeline for hazard identification. Chemicals were tested with 19 concentrations (*n* = 1) up to 200 µM, in the presence and absence of Aroclor 1254-induced rat liver S9. To identify clastogenic chemicals, %MN values at each concentration were compared to a distribution of batch-specific solvent controls; this was followed by cytotoxicity assessment and benchmark concentration (BMC) analyses. The approach classified 157 substances as positives, 25 as negatives, and 110 as inconclusive. Using the approach described in Bryce et al. (Environ Mol Mutagen 52:280–286, 2011), we identified 15 (5%) aneugens. IVIVE (in vitro to in vivo extrapolation) was employed to convert BMCs into administered equivalent doses (AEDs). Where possible, AEDs were compared to points of departure (PODs) for traditional genotoxicity endpoints; AEDs were generally lower than PODs based on in vivo endpoints. To facilitate interpretation of in vitro MN assay concentration–response data for risk assessment, exposure estimates were utilized to calculate bioactivity exposure ratio (BER) values. BERs for 50 clastogens and two aneugens had AEDs that approached exposure estimates (i.e., BER < 100); these chemicals might be considered priorities for additional testing. This work provides a framework for the use of high-throughput in vitro genotoxicity testing for priority setting and chemical risk assessment.

**Supplementary Information:**

The online version contains supplementary material available at 10.1007/s00204-022-03286-2.

## Introduction

In many countries, legislation is in place requiring genotoxicity testing before new chemicals can be manufactured or imported above a certain volume or based on intended use. This poses a challenge as the rate of new chemicals production for commercial and industrial use continues to increase. Traditional in vitro genotoxicity testing tools such as the Ames assay, Comet assay and Micronucleus test, along with in vivo testing on animals, lack the necessary throughput to maintain pace with the emergence of new chemicals. An additional challenge is the lack of such data for the large numbers of chemicals that already exist on the market (Brendt et al. 2021; Shibai-Ogata et al. 2011). Moreover, the practice of risk assessment is moving towards reducing the use of animals (Krewski et al. 2020; Liu et al. 2019; Scholz et al. 2013; Wallace Hayes et al. 2020). Thus, genotoxicity testing and assessment would benefit from new in vitro approaches that can more efficiently test, classify, and prioritize chemicals for evaluation and regulation.

The ToxCast project of the Center for Computational Toxicology and Exposure (CCTE) at the U.S. Environmental Protection Agency (US EPA) aims to prioritize and assess hazard for environmentaol chemicals by applying screening technologies that are more cost-effective and higher-throughput than conventional tests (Knight et al. 2009). The datasets generated from these high-throughput screening assays are analyzed and modeled by novel computational approaches to predict potential chemical toxicities to human physiologies. The ToxCast framework has been implemented in phases (Dix et al. 2007; Kavlock et al. 2012; Kligerman et al. 2015). Phases I and II tested chemicals that had extensive toxicity data (primarily pesticides) and potential endocrine-related activities, respectively. Phase III added new assays and endpoints, as well as new chemicals, including endocrine disruptors, flame retardants, and mixtures. However, the number of assays in ToxCast that assess genotoxicity are minimal (<1%) and these tests are known to lack sensitivity (approximately 13%) for this endpoint (Hsieh et al. 2019). Thus, alternative and more sensitive approaches are needed to support the identification of genotoxic hazards as part of high-throughput screens.

The in vitro micronucleus (MNvit) test is one of the most sensitive and widely used tests for genotoxicity (Decordier and Kirsch-Volders 2006; Kirsch-Volders et al. 2011). The test detects DNA fragments or extra chromosomes that are caused by clastogenic and aneugenic mechanisms, respectively. These chromosomes or chromosome fragments manifest as observable micronucleus events in the cytoplasm of the daughter cells in interphase (Decordier and Kirsch-Volders 2006; Fenech 2000; OECD 2016). In a MNvit test, cells are treated with a chemical, and micronucleus frequencies are measured against solvent control treated cells (i.e., controls). Traditionally, micronucleus frequencies have been measured by manually counting micronuclei in interphase spreads using microscopy, which can be very time-consuming. In the past decade or more, automated flow cytometric methods have become increasingly popular for micronucleus analysis. The Organisation for Economic Co-operation and Development (OECD) test guideline indicates a positive response for MNvit when: (a) at least one of the test concentrations (of a minimum of three recommended plus controls) exhibits a statistically significant increase compared with the concurrent negative control; (b) a trend-test demonstrates that the increase is concentration-related in at least one experimental condition; and (c) the positive results are outside the distribution of the historical negative control data (OECD 2016).

The MNvit test is typically conducted in six-well plates and consists of at least three concentrations plus controls in duplicate (OECD 2016). A low or non-cytotoxic chemical is usually tested with concentrations that are at two- to three-fold intervals. Chemicals are generally tested at non-cytotoxic, moderately cytotoxic and cytotoxic concentrations, where the maximum concentration seeks to achieve 55 ± 5% cytotoxicity. In the absence of cytotoxicity, a top concentration of 10 mM (or 2 mg/mL) or the highest soluble concentration is recommended. As presently conducted according to the OECD-compliant protocol with a limited concentration range, the MNvit test is not easily integrated into the current ToxCast framework where testing is conducted using 96- or 384-well plate formats across a broad concentration range. Here, MNvit testing was conducted using automated flow cytometry of 19 chemical concentrations to a maximum of 200 µM (*n* = 1 per concentration) alongside solvent controls, making it feasible to integrate the MNvit assay with the standard battery of tests conducted within the ToxCast framework. Moreover, this study design is more suited to application of the benchmark concentration (BMC) approach for assessment of chemical potency. Specifically, BMC precision is augmented by distribution of the experimental replicates to as many concentrations as possible (Slob et al. 2005; Woutersen et al. 2001).

Applying BMC analyses to MNvit datasets for potency comparisons and prioritization is aligned with initiatives to move away from using genotoxicity testing exclusively for dichotomous hazard classification (positive/negative) (White et al. 2020). Indeed, genotoxicity endpoints are increasingly being recognized as *bona fide* toxicological endpoints for risk assessment and regulatory decision making (MacGregor et al. 2015; White and Johnson 2016; White et al. 2020). To use in vitro data in risk assessment applications, it is necessary to apply in vitro to in vivo extrapolation (IVIVE) models to derive administered equivalent doses (AEDs). AEDs, based on IVIVE and toxicokinetics models, provide human dose context to in vitro (geno)toxicity data by determining the correlation between intake dose and chemical disposition to various body compartments (Honda et al. 2019). Specifically, the AED is the estimated dose required to reach an internal concentration in the plasma that is equivalent to the concentration seen to be genotoxic using in vitro assays, and AEDs can be viewed as surrogate points of departure in the absence of in vivo data*.* Therefore, AEDs provide a more relevant potency ranking of chemical hazard than what is achieved using in vitro toxicity concentrations alone (Rotroff et al. 2010; Wetmore et al. 2012).

AEDs can be compared to human exposure values to derive bioactivity exposure ratios (BERs). BERs are analogous to the traditional margin of exposure used in risk assessment in that chemicals with a lower BER possess a higher potential for risk. For example, recent work has demonstrated the utility of in vitro BER data and IVIVE in establishing points of departure (PODs) that are protective (i.e., conservative) relative to traditional and apical PODs (Beal et al. 2022; Gannon et al. 2019; Paul Friedman et al. 2020). Furthermore, this work also demonstrates how BERs may be used in a tiered risk assessment framework. As a follow-up, Health Canada prepared a case study evaluating the BERs of 41 compounds with previous risk assessments conducted under the Chemicals Management Plan, and this work determined that a BER of less than 100, or a log_10_BER of less than 2, identified all six of the non-genotoxic compounds classified as toxic under the Canadian Environmental Protection Act (Health Canada 2021). Thus, BER data can be a useful tool for prioritizing chemicals for further work based on non-genotoxic endpoints, including risk assessment as relevant. However, the application of HTTK models for IVIVE of in vitro genotoxicity data to derive BERs has not yet been explored.

In this study, we analyzed the MNvit datasets of 292 chemicals from ToxCast and applied our extensive experience with this assay to produce a pipeline to evaluate the potential genotoxicity of the agents tested. Our proposed decision-making scheme classifies a chemical as positive, negative, or inconclusive by extensively evaluating the relative survival, non-parametric concentration–response trends, and BMC values for MN induction. This large dataset provides a unique opportunity to study the application of toxicokinetics to in vitro genetic toxicology data to determine the relationship between our high-throughput MNvit AEDs and PODs from traditional in vivo genetic toxicology and cancer studies. We also derived BERs to support quantitative evaluation of the tested compounds. BERs serve to identify the compounds with the highest potential for concern (i.e., AEDs approaching human exposure levels), making them priorities for further examination in scoping and risk assessment activities. We envision that this modernized approach can be used to complement test batteries applied in tiered testing strategies (Thomas et al. 2013, 2019), increasing the robustness of genotoxicity assessment, as well as accelerating the rate of chemical risk characterization.

## Methods

### Chemical selection

Chemicals were selected for testing by cross-matching samples available in sufficient quantity from existing ToxCast chemical libraries and inclusion in available databases or literature providing genotoxicity information (Richard et al. 2016). The genotoxicity data sources included the Chemical Carcinogenesis Research Information System (https://www.nlm.nih.gov/databases/download/ccris.html), the Priority-based Assessment of Food Additives database (Benz and Irausquin 1991), the Carcinogenic Potency Database (https://www.nlm.nih.gov/databases/download/cpdb.html), The National Toxicology Program’s genotoxicity data in the Chemical Effects in Biological Systems database (ftp://anonftp.niehs.nih.gov/ntp-cebs/datatype/GENETOX_Genetic%20Toxicology%20-%20Micronucleus/), genotoxicity summary of food additives in Japan (Yamada and Honma 2018), and the Benigni/Bossa rulebase for mutagenicity and carcinogenicity data used in Toxtree (Benigni et al. 2008). There were 292 chemicals selected that had sufficient quantity for testing, structural features (i.e., chemotypes) indicating genotoxic potential, and/or, previous investigations of genotoxicity. Additional candidates beyond the 292 chemicals were identified but testing of these chemicals was beyond the time and funds allocated to this project.

### MNvit experimentation and datasets

Experiments with 292 chemicals, and their concurrent DMSO solvent controls, were performed by BioReliance, and MNvit datasets were prepared by the US EPA. The MN assay dataset contains 79 files in Microsoft Excel format. Each file contains data generated from two 96-well plates, with or without the addition of S9. A total of 292 chemicals were tested in the MN assay, and some of the chemicals were tested in more than one plate, for a total of 614 tests. The assays were performed on 18 different days, and this was used to generate batch-specific control 95^th^ percentile reference values.

All agents were tested in Chinese hamster ovary (CHO) cells obtained from the ATCC (American Type Culture Collection) (Manassas, VA). CHO cells were cultured in complete medium: Ham’s F-12 medium, supplemented with 10% fetal bovine serum, 100 units penicillin/mL and 100 µg streptomycin/mL. Cells were prepared in T75 flasks. The culture medium was aspirated and cells with 60–90% confluence were washed once with calcium and magnesium-free phosphate buffered saline. The cells were trypsinized with 2 mL of 0.1% trypsin for approximately 4 min at 37 °C. After the cells were detached, the flasks harboring cells were diluted with medium to achieve a final density suitable to 96-well format: 5 × 10^4^ cells/mL. A volume of 0.2 mL of the cell suspension was added to each well of the 96-well plates for chemical exposure with and without S9. Cultures were incubated at 37 °C for 16–24 h before treatment. Cells were tested with 19 concentrations (4.50, 5.63, 7.04, 8.80, 11.0, 13.7, 17.2, 21.5, 26.8, 33.6, 41.9, 52.4, 65.5, 81.9, 102, 128, 160, and 200 µM) for 24 h in the absence of rat liver S9 at 37 °C. In the S9-activated study, cells were treated for 3–4 h in the presence of Aroclor 1254-induced rat liver S9 at 37 °C, washed, and then incubated in test article-free complete medium at 37 °C for 24 h. Replicate vehicle controls (1% DMSO) were included on each plate. Three concentrations of a standard genotoxicant, vinblastine (0.025, 0.0125, and 0.00625 μg/ml) or cyclophosphamide (5.0, 2.5 and 1.25 μg/ml), were tested in parallel as positive controls without and with activation systems, respectively. In total, eight wells of negative controls (DMSO), six wells of positive controls (vinblastine − S9 or cyclophosphamide + S9) and four chemicals, each having 19 concentrations plus a control, were applied to each plate. Following the treatments, the treatment medium was aspirated and the plates were placed on ice for 20 min.

The flow cytometry-based MNvit and cytotoxicity assay was performed using the MicroFlow^®^ kit (Litron Laboratories, Rochester, New York, USA) with more details related to protocol and compositions of buffers/solutions described previously (Avlasevich et al. 2006). A brief description of the MicroFlow methods is provided here. In each well of the plate, 50 µL of Nucleic Acid Dye A solution was added, and the plates were placed under a white fluorescent light (approximately 15 cm away). Samples were exposed to visible light for 30 min while on ice. Approximately 150 µL of 1X Buffer solution with 2% Fetal Bovine Serum was added to each well and the Dye/Buffer was then aspirated. Afterwards, 100 µL of Complete Lysis Solution I containing RNase and Nucleic Acid B (SYTOX Green) was added to each well, and the plates were incubated at 37 °C for 30 min, followed by 30 min at room temperature with shaking at 200–300 rpm. Complete Lysis Solution II, containing counting beads and Nucleic Acid B (SYTOX Green), was added to each well (100 µL), and plates were incubated for 30 min at room temperature. The plates were stored at 2–8 °C for up to 3 days prior to flow cytometry.

Micronucleus frequency, nuclei to beads ratio (i.e., relative survival) and hypodiploid events in treated cells were assessed using a BD FACSCanto II flow cytometer with 488 nm excitation laser. Samples were equilibrated at room temperature for approximately 30 min, and then the solvent control cell suspension was transferred to a 5 mL flow cytometry tube and analyzed on the cytometer. Unwanted events, including debris and apoptotic or necrotic events, were removed by gating out the regions containing these events (see Fig. 1 of Avlasevich et al. 2006 as an example). Micronucleus values were expressed as a percent frequency by dividing the number of events within the micronucleus-gated region by the number of events within the nucleated region and multiplying by 100. Hypodiploid nuclei were gated during flow cytometric analysis with the percentage frequency used to identify aneugenicity. Relative survival rate was obtained by dividing the nuclei to bead ratio in the sample by the ratio of the vehicle control and multiply by 100 (Avlasevich et al. 2006).

**Fig. 1:**
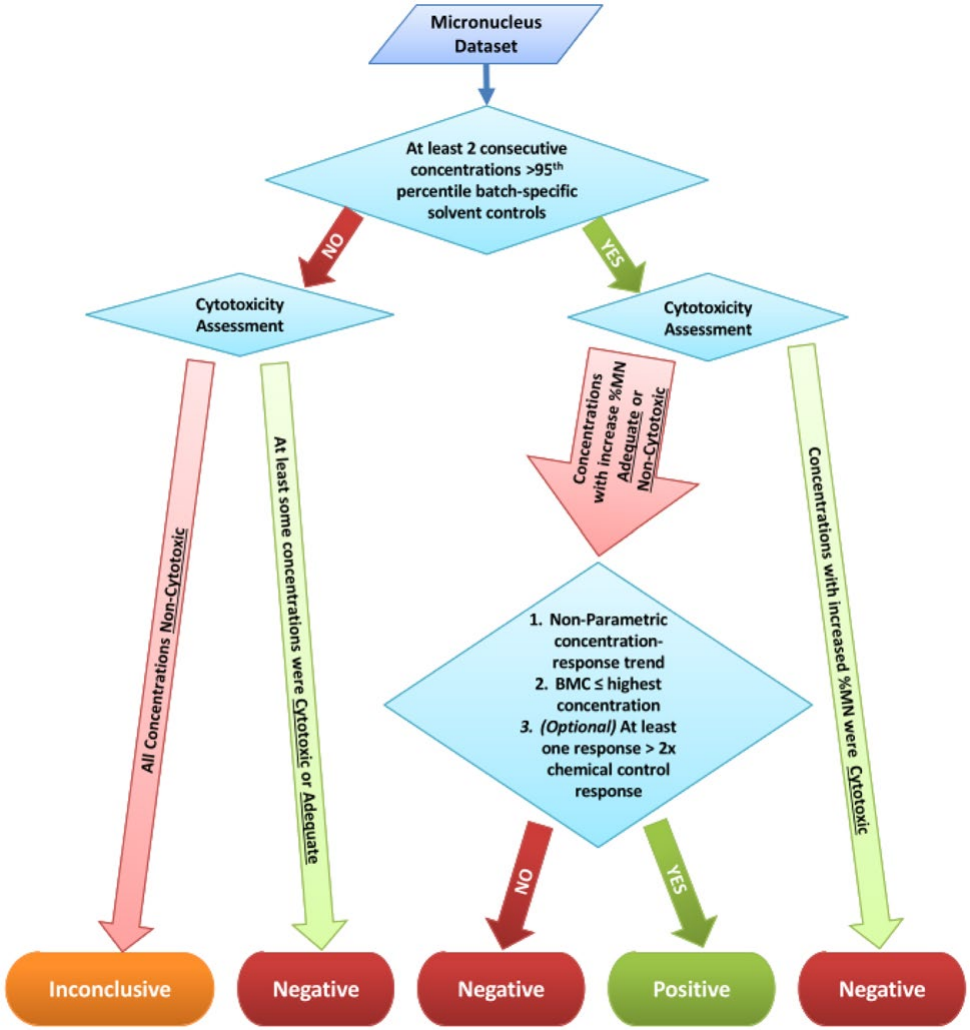
Decision-making scheme for evaluating MN assay results (color figure online)

Counts of micronucleus and hypodiploidy events were reported as percentages relative to the control concentration’s nucleated events. Fold-changes are the ratio of each concentration’s micronucleus or hypodiploidy percentage over that of the DMSO control.

### Data preparation and analysis

In vitro datasets were downloaded in Microsoft Excel formats, with each plate as an individual file. An open-source software, XLS to CSV Converter, (https://cwestblog.com/2013/04/12/batch-excel-to-csv-converter-application/) was used to convert each MS Excel sheet and file into a tab-delimited text file. Perl scripts were created to separately pool control datasets and treatment datasets from each plate for further analyses.

Data analysis was performed using the R Project for Statistical Computing (https://www.r-project.org/) under R Studio (https://www.rstudio.com/). Non-parametric trend tests to determine concentration–response were applied using the Jonckheere test function (https://www.rdocumentation.org/packages/clinfun/versions/1.0.14/topics/jonckheere.test) of the *Clinfun* package in the R statistics environment (Seshan 2018).

### Data quality assessment

Solvent control (DMSO) and positive controls (cyclophosphamide and vinblastine) were separately parsed and pooled to individual files. Data were imported into R for analysis and assessment. For positive control datasets, plots of MN frequency against relative survival rates were generated for each treatment concentration, 1.25, 2.5 and 5 µm for cyclophosphamide (+ S9) and 0.00625, 0.0125 and 0.025 µm for vinblastine (− S9), to identify distributions and outliers. For solvent controls, four histograms were plotted for MN and hypodiploidy frequencies, in the presence or absence of S9, to identify outliers. 95th percentile values for MN and hypodiploidy frequencies were computed for each batch date. Using this approach, one plate was identified as an outlier and removed, i.e., 20131009 Plate 2.

### Benchmark concentration (BMC) analysis

BMC modeling was conducted using the PROAST package (version 61.5) for the R statistical environment. PROAST was selected for BMC analysis because it is capable of modeling BMC without replicates. The benchmark response (BMR) for %MN and %hypodiploid was based on first calculating one standard deviation (SD) of the mean control values, excluding the outlier, noted above. One SD was an approximate 30% and 60% change in %MN and %hypodiploid, relative to control, respectively. Thus, BMR values of 30% and 60% increases were used for %MN and %hypodiploid. For each dataset (i.e., chemical ± S9), concentration–response modeling was performed on concentrations categorized as ‘non-cytotoxic’ and ‘adequate’ (described in more detail below) using the model 5 (Hill and Exponential) model families, based on the European Food Safety Authority (EFSA) Science Committee’s recommendations for continuous data (EFSA 2009, 2017). Model selection was informed using the Akaike Information Criterion (AIC). For each compound, we also retained information describing the two-sided 90% confidence interval (e.g., bounded by the BMC lower limit (BMCL) and BMC upper limit (BMCU)).

### Micronucleus assessment

A decision-tree approach was developed to assess MN based on %MN against batch-specific solvent controls, cytotoxicity assessments, and concentration–response tests (Fig. 1). Within this decision tree, cytotoxic refers to concentrations at which relative survival was < 40%; adequate refers to levels of cytotoxicity between 40–60% (i.e., a requirement in the OECD MNvit test guideline, unless the top concentration tested is 10 mM, 2 mg/mL or 2 µL/mL); and non-cytotoxic refers to test concentrations with relative survival > 60%.

A positive assessment for MN required: (a) that two consecutive concentrations with a %MN greater than their respective ± S9 95th percentile of batch-specific solvent controls; (b) that the observed increase in %MN occurred at non-cytotoxic or adequate cytotoxicity levels; (c) a concentration–response trend, which was assessed using a non-parametric test; and (d) a BMC (BMR30) value that was lower than or equal to the highest concentration used in BMC modeling. We also assessed the outcome if an additional filter requiring at least a two-fold increase above controls for at least one concentration was also applied, i.e., as a more conservative approach. If conditions (a) and (b) were met, but there was no concentration–response by the non-parametric test (i.e., condition (c)), or the chemical had a BMC > highest test concentration [i.e., condition (d) whereby the model suggested that the chemical did not induce the BMR within the given test concentrations], the assessment was classified as negative. When a %MN increase was observed, but only within overtly cytotoxic concentrations (i.e., relative survival rate < 40%), the chemical was also classified as negative.

Negative calls were made if the chemical did not have at least two consecutive %MN above the 95th percentile of controls, and the top concentration tested achieved the minimal required level of cytotoxicity (i.e., either ‘cytotoxic’ or ‘adequate’). In cases where the test did not achieve the minimum specified top threshold for cytotoxicity, the negative result was considered inconclusive.

### Hypodiploidy (aneugenicity) assessment

Two approaches were used to identify agents that induce hypodiploidy. In the first approach, the decision scheme outlined in Fig. 1 was applied, but the %MN was replaced with %hypodiploid and a BMR60 was used. The second approach applied the method suggested by Bryce et al. (2011). In this approach, a positive hypodiploidy call was made if fold change relative to concurrent controls in %MN was greater than three, and fold change for %hypodiploid cells was greater than ten. In this approach, the fold-change values were directly obtained from the datasets obtained from EPA and the call is either positive or negative.

### Derivation of administered equivalent doses (AED)

IVIVE modeling of BMC in µM to determine AEDs in mg/kg bw/day was performed using the HTTK package v1.10 (Pearce et al. 2017) in R following methods used previously (Beal et al. 2022; Paul Friedman et al. 2020). Specifically, the three compartment steady-state model (“3compartmentss”), modified from Wetmore et al. (2012, 2015); Wetmore (2015), was used to calculate the steady-state concentration in the plasma (*C*_*ss*_) at a constant infusion dose rate of 1 mg/kg bw/day. Assuming a linear relationship, IVIVE can be used to model the dose rate (i.e., AED) required to reach an internal *C*_*ss*_ equal to the BMC shown to be genotoxic in the CHO cells. The AED was derived using the formula:

$${\text{AED}} = {\text{benchmark concentration}}\;\left( {\mu {\text{M}}} \right) \times \frac{{1\frac{{{\text{mg}}}}{{{\text{kg}}}}}}{{C_{ss} \left( {\mu {\text{M}}} \right)}}$$

The calc_mc_css() function in HTTK was used to model the *C*_*ss*_ using parameters well.stirred.correction = TRUE and output.units = “µM”. To run the 3compartmentss model and return units in mg/kg bw/day, the in vitro parameters consisting of intrinsic clearance (*Cl*_int_) and fraction unbound in the plasma protein (*F*_up_) were needed. In addition, the physico-chemical properties consisting of molecular weight and the octanol/water partition coefficient (log *P*) were also required. These data were unavailable for many of the compounds tested in this study; therefore, in silico predictions were used to obtain predictions for these parameters (Supplemental File 1). Previous work has demonstrated that ADMET Predictor can provide reliable estimates of in vitro parameters for HTTK modeling to derive stable estimates of *C*_*ss*_ (Pradeep et al. 2020). Thus, ADMET Predictor version 9.5 was used to provide *F*_up_ percentage (hum_fup%) and human liver microsomal clearance (CYP_HLM_CLint) by CASRN (CAS number). CYP_HLM_CLint (µL/min/mg) were adjusted to *Cl*_int_ HTTK units (µL/min/10^6^ cells) by dimensional analysis using scaling factors (Barter et al. 2007) that have been previously applied (Sipes et al. 2017):

$$C{l}_{\mathrm{int}}=\mathrm{CYP}\_\mathrm{HLM}\_\mathrm{CLint}\times \frac{32\; \mathrm{mg}\; \mathrm{of}\; \mathrm{microsomal} \;\mathrm{protein}}{\mathrm{g}\; \mathrm{of}\; \mathrm{liver}}\times \frac{1 \;\mathrm{g}\; \mathrm{of}\; \mathrm{liver}}{99 \times {10}^{6} \;\mathrm{cells}}$$

The ChemmineOB R package (Horan and Girke 2020), was used to interface with the OpenBabel C + + project (O’Boyle et al. 2011) to provide molecular weight and log *P* estimates for each chemical. The required parameters for IVIVE were imported into HTTK using add_chemtable() function. The overwrite parameter was set to FALSE to prioritize any existing experimental data in the HTTK database over the supplemented in silico parameters.

### Comparison of administered equivalent doses to traditional in vivo PoDs

Traditional in vivo genotoxicity data were compared against AEDs to build confidence in the developed workflow and the utility of AEDs in the context of risk assessment. Specifically, traditional in vivo PODs in mg/kg bw/day were extracted from the ToxValDB, which is a highly-structured database containing cancer and genotoxicity data in addition to many other endpoints (Williams et al. 2017). The AEDs derived in this study for all of the compounds classified as positive clastogens or aneugens in MN assay were compared against the traditional PODs where the study type is listed as “cancer” or “genetox.”

### Determination of bioactivity exposure ratios (BERs)

BERs were determined using the most recent ExpoCast exposure estimates (Cohen Hubal et al. 2010; Wambaugh et al. 2013) downloaded from the CompTox Chemicals Dashboard (Williams et al. 2017) on October 22, 2020. Both the median and 95th percentile exposure estimates were used to derive BERs. BERs were reported as the log_10_BER on the logarithmic scale (log_10_AED − log_10_Exposure), and as BER on the arithmetic scale (AED/Exposure).

## Results

### Data quality assessment using positive controls

**Fig. 2:**
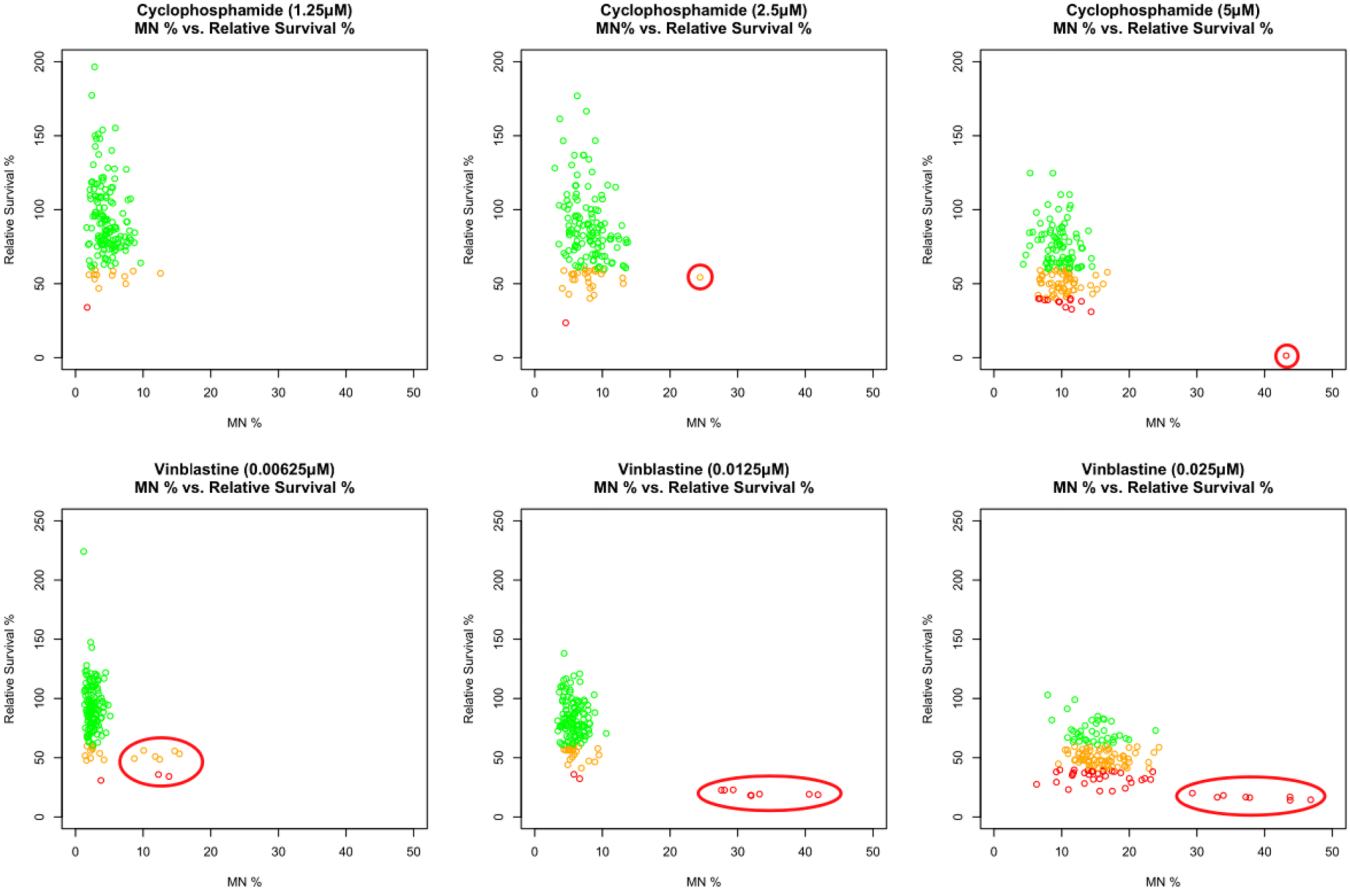
Scatter plots of positive controls, vinblastine (− S9) and cyclophosphamide (+ S9), versus relative survival rate. Each plot represents increasing concentrations of test agents (1.25, 2.5, and 5 µM for cyclophosphamide, and 0.00625, 0.0125, and 0.025 µM for vinblastine). Wells with relative survival rates below 40% are colored in red, between 40 and 60% in orange, and above 60% in green. One outlier was identified for cyclophosphamide for each of 2.5 µM and 5 µM and eight outliers were identified for all three vinblastine concentrations (circled in red). Two outliers were not plotted for cyclophosphamide at 1.25 µM (color figure online)

Positive controls, cyclophosphamide with S9 and vinblastine without S9, were pooled according to their concentrations, and scatter plots were generated of %MN against their respective relative survival rates (Fig. 2). In general, positive controls behaved as expected demonstrating declines in relative survival and increases in %MN with increased variance in %MN as concentration increased. Of the 158 data points in each concentration, one outlier (0.6%) was identified for cyclophosphamide for each of 2.5 µM and 5 µM. For vinblastine, eight (5%) outliers were identified for all three concentrations. These all belong to the same date batch (*20130903 plates 1, 2, 3, and 4*), indicating a potential batch effect only for vinblastine. Overall, no clear plate-effects were observed.

## Data Quality Assessment Using Solvent Controls

**Fig. 3:**
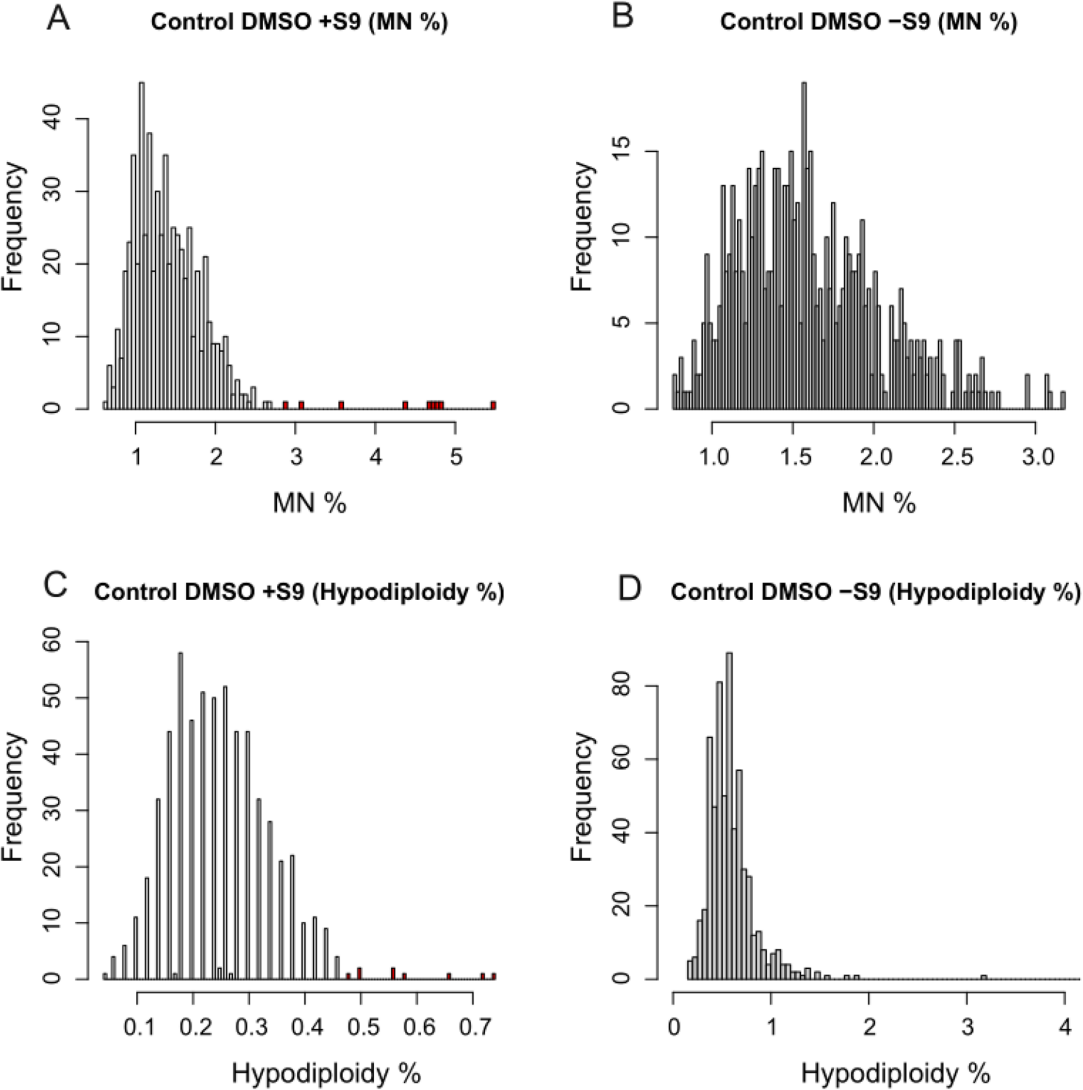
Overall distributions for solvent control (DMSO) %MN and %hypodiploid, ± S9. For the %MN and %hypodiploid + S9 distributions (**A** and **C**), nine outliers belonging to 20131009 Plate 2 are colored in red. Four data points from solvent control DMSO − S9 (hypo %) (**D**) were also removed (hypodiploid ranges from 15 to 132%). No clear outliers were observed in (**B**) (color figure online)

Solvent controls (DMSO) were pooled from each plate and grouped into DMSO − S9 and DMSO + S9 to investigate distributions and outliers (Fig. 3). In the − S9 %hypodiploid analysis, four outliers were observed at 3.2%, 15.5%, 28.3%, 61.8%, and 132.5%, the latter four were removed from the figure; the respective plates were *20130911 Plate 5, 20130923 Plate 5a, 20130911 Plate 6, 20130923 Plate 5a, and 20130923 Plate 5a*. A plate effect was observed in the + S9 distributions for both %MN and %hypodiploid cells (colored in red). Further investigations revealed that *20131009 Plate 2* contributed to these outliers. These outliers were subsequently removed from further analysis.

One of the criteria to assess MN is to compare the %MN of a chemical at every concentration to reference values generated from batch-specific solvent controls. To compute these reference values, we pooled the control datasets within dates. Each date was considered a batch, and their control %MN and %hypodiploidy frequencies were used to compute the 95th percentile values (i.e., 95% confidence interval of the population) (Table 1). This revealed that %MN for − S9 and + S9 ranged from 1.65% to 2.96%, and from 1.22% to 2.31%, respectively.

**Table 1:** Upper 95th percentiles of the distribution of solvent control (DMSO) %MN and %hypodiploid (%hypo) frequencies calculated based on the distributions of DMSO datasets per batch (date—year, month, day)

Batch date	%MN 95th percentile		%Hypodiploidy 95th percentile	
	− S9	+ S9	− S9	+ S9
20130827	1.86	1.22	0.84	0.30
20130829	1.80	1.75	0.64	0.38
20130903	2.31	1.81	0.56	0.32
20130905	1.92	2.00	0.61	0.42
20130906	2.31	2.29	0.81	0.37
20130909	2.47	2.24	0.66	0.39
20130911	2.96	2.10	1.46	0.36
20130916	2.65	2.06	0.76	0.37
20130918	2.47	2.24	0.82	0.44
20130923	2.28	1.99	0.80	0.41
20130925	2.21	2.31	0.77	0.38
20130930	2.36	2.12	1.36	0.49
20131007	2.19	2.12	1.03	0.44
20131009	2.46	2.00^a^	0.75	0.32^a^
20131015	1.65	1.61	0.93	0.54
20131017	1.84	1.36	0.74	0.32
20131024	2.28	2.00	0.70	0.41

^a^Data collected on “20131009 Plate 2” were removed from the calculations

### Relative survival rate and cytotoxicity assessment

Cytotoxicity for each chemical, concentration, and S9 treatment, was evaluated using its relative survival data (Supplemental File **2**). Relative survival rates below 40%, between 40 and 60%, and above 60% were classified as cytotoxic, adequate, and non-cytotoxic, respectively. Of the 11,570 unique data points, 571, 274, and 10,725 were classified as cytotoxic, adequate, and non-cytotoxic, respectively. Among all chemical treatments, 56%, 28.4%, 13%, and 2.6% had a relative survival rate < 100%, 100–120%, 121–150%, and > 150%, respectively. In the positive controls, 80%, 12.6%, 5.4% and 2% for cyclophosphamide + S9, and 85%, 11.8%, 3% and 0.2%, for vinblastine − S9, had relative survival rates of < 100%, 100–120%, 120–150%, and > 150%, respectively.

### BMC analysis

BMC30 and BMC60 values were generated for each dataset (± S9) for MN and hypodiploid frequencies, respectively. Of the 614 datasets, 284 did not produce a BMC30 value for MN (i.e., had no concentration–response or had a BMC > highest dose) and 311 did not enable calculation of a BMC60 value for hypodiploidy. None of the BMC30 values for MN exceeded the highest concentrations included during BMC modeling, whereas five BMC60 values for hypodiploidy exceeded the highest concentrations used.

### Micronucleus assessments

We performed MN assessment according to the decision-making scheme outlined in Fig. 1. Using our criteria, 180 data sets yielded positive results, either in the presence or absence of S9 (Table 2; further details in Supplemental Files 3 and 4). This amounts to 157 of the 292 chemicals with a positive MN response in at least one of two MN tests (± S9), and thus classified as clastogenic. In contrast, 25 chemicals were negative in both the presence and absence of S9. The remaining 110 chemicals were categorized as inconclusive; these chemicals either yielded inconclusive calls in both ± S9 MN tests, or were inconclusive in one and negative in the other MN test. Note that each chemical is found only once in the ‘Total number of unique chemicals’ row (for a total of 292 chemicals), reflecting that each chemical was given a single positive, negative or inconclusive call. Of the 157 positive chemicals, 38 were MN positive only in the presence of S9, indicating that these chemicals require metabolic generation of a clastogenic metabolite.

**Table 2:** Hazard classifications based on %MN assessments following the decision tree (Fig. 1)

MN assessment	Positive	Negative	Inconclusive
# of Datasets			
− S9	115 (77)^a^	114 (152)	79 (79)
+ S9	65 (56)	83 (92)	158 (158)
Total	180 (133)	197 (244)	237 (237)
Total # of unique chemicals	157 (115)^P^	25 (41)^N^	110 (136)^I^

^a^Numbers in brackets are chemicals assessed with an additional filter of at least one response greater than two-fold above the solvent control

^P^‘Positive’ chemicals had positive calls in either ± S9 tests

^N^‘Negative’ chemicals had negative calls in both ± S9 tests

^I^‘Inconclusive’ chemicals had either (a) inconclusive calls in both ± S9 tests; (b) inconclusive calls in one of the ± S9 tests and negative in the other

We also applied an additional filter of at least one response greater than two-fold above the DMSO solvent control (i.e., concentration of zero µL/mL) prior to modeling to derive a BMC, as in vitro chromosome damage assays are often considered overly sensitive (i.e., low specificity). With this filter, the numbers of chemicals classified as positive, negative and inconclusive were 115, 41 and 136, respectively.

To identify chemicals causing hypodiploidy (i.e., aneugens), we first applied an identical decision-making scheme. This analysis yielded 209 chemicals with hypodiploidy positive results in either the presence or absence of S9, and 27 and 56 chemicals with negative and inconclusive calls, respectively (Table 3; with further details in Supplemental File 3). As a comparison, we also applied a more stringent approach to the identification of aneugens (Bryce et al. 2011). Using this approach, 15 chemicals were hypodiploidy positive and 277 were negative, either in the presence or absence of S9 (Table 4). Of the 15 chemicals identified as hypodiploid positive, there was literature to support that ten of them are aneugenic (Supplemental File 4). Two chemicals, Amiodarone hydrochloride (CAS# 19774-82-4) and Michlers ketone (CAS# 90-94-8) were positive only in the presence of S9, indicating the requirement of metabolic activation to generate an aneugenic metabolite. Nine of the 15 aneugenic positive chemicals according to Bryce et al. (2011) were also classified as clastogenic in our proposed decision-tree approach, regardless of the two-fold change filter.

**Table 3:** Hypodiploidy (aneugenicity) hazard classification using the same decision tree as shown in Fig. 1, but replacing 95th percentile for %MN with the 95th percentile for %hypodiploid cells

Hypodiploidy assessment #1	Positive	Negative	Inconclusive
# of datasets			
− S9	193 (154)^a^	89 (128)	26 (26)
+ S9	34 (31)	79 (82)	193 (193)
Total	227 (185)	168 (210)	219 (219)
Total # of unique chemicals	209(172)	27 (35)	56 (85)

^a^Numbers in brackets are chemicals assessed with an additional filter of at least one response greater than two-fold above the solvent control

**Table 4:** Hypodiploidy (aneugenicity) hazard assessment using the classification method described by Bryce et al. (2011)

Hypodiploidy approach 2^a^	Positive	Negative
# of datasets		
− S9	13	295
+ S9	5	301
Total	18	596
Total # of unique chemicals	15	277

^a^Positive if MN fold change > 3 and hypodiploidy fold-change > 10

### Comparison of AEDs and PODs

BMC confidence interval plots for both %MN and %hypodiploidy for each chemical are rank ordered in Supplemental Files 5, 6 and 7. AEDs could be derived for 137 of the compounds exclusively classified as positive for clastogenicity using the decision-tree approach (i.e., without the optional two-fold filter), and 14 of the chemicals classified as aneugens using the Bryce et al. 2011 approach (Supplemental File 1) based on availability of the necessary kinetics data and models for IVIVE. For AED determination, the lower BMC ± S9 was used. These in vitro AEDs are referred to as POD_Clastogen_ and POD_Aneugen_.

To compare traditional PODs and AEDs directly, genotoxicity and cancer study data were extracted from ToxValDB. The sources of the POD data were from the: (a) European Union COSMOS project for 111 PODs, (b) European Chemicals Agency (ECHA) or ECHA International Uniform Chemical Information Database (IUCLID) for 45 PODs, and (c) Health Assessment Workplace Collaborative (HAWC) for 16 PODs. There were a total of 172 in vivo PODs for 33 chemicals classified as clastogenic using the decision-tree approach. Specifically, there were 109 PODs from cancer studies for 31 chemicals (referred to as POD_Cancer_) and 63 PODs from in vivo genotoxicity studies for 12 chemicals (referred to as POD_Genetox_). None of the chemicals positive for hypodiploidy had data in ToxValDB and therefore, comparisons could only be made between AEDs and traditional PODs for clastogens.

**Fig. 4:**
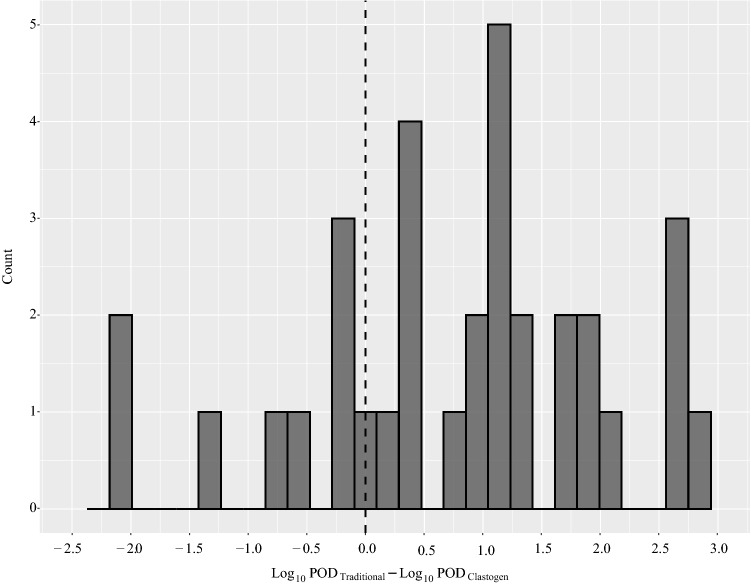
POD ratio distribution between lowest traditional POD (i.e., across in vivo genotoxicity and cancer studies) and AEDs determined herein using in vitro MN data. The dashed line indicates where the traditional POD and AED are equivalent. Chemicals to the left of the dashed line have a higher AED than the traditional POD, and chemicals to the right have a lower AED than the traditional POD (i.e., are more conservative)

When using the lowest traditional POD from both genotoxicity and cancer studies, the AED was lower than the traditional POD for 24 out of 33 (72.7%) of possible comparisons (Fig. 4). The log_10_Traditional POD − log_10_AED difference ranged from − 2.10 to 2.82 (Supplementary File 8). The average difference was 0.77 (i.e., AED was 5.9-fold lower than traditional POD on arithmetic scale). There were only two compounds where the difference was below − 2 (i.e., AED is 100-fold higher than traditional POD on an arithmetic scale). Folic acid and acrylamide both had a log10 difference of − 2.10. The traditional POD for folic acid was based on in vivo micronucleus data with a highest no effect level of 0.0033 mg/kg bw/day, and the lowest POD for acrylamide was from a cancer study with a no-observed-adverse-effect level (NOAEL) of 0.1 mg/kg bw/day.

When the analysis was limited to the lowest POD from cancer studies, the AED was lower than the traditional POD for 26 out of 31 (83.9%) compounds with available cancer data. The difference ranged from − 2.10 to 3.06, with an average difference of 1.15 (i.e., AED is 14.2-fold lower on arithmetic scale). When the analysis was limited to the lowest POD from in vivo genotoxicity studies, the AED was lower than the traditional POD for 8 out of 12 (66.7%) of compounds with data. The difference ranged from − 2.10 to 2.82, with an average difference of 0.42 (i.e., AED is 2.6-fold lower on arithmetic scale). Thus, the AED is typically lower than the cancer POD by one order of magnitude and is on the same order of magnitude for in vivo genetic toxicity PODs.

### Ranking of potential chemical risk based on bioactivity exposure ratios (BERs)

**Fig. 5:**
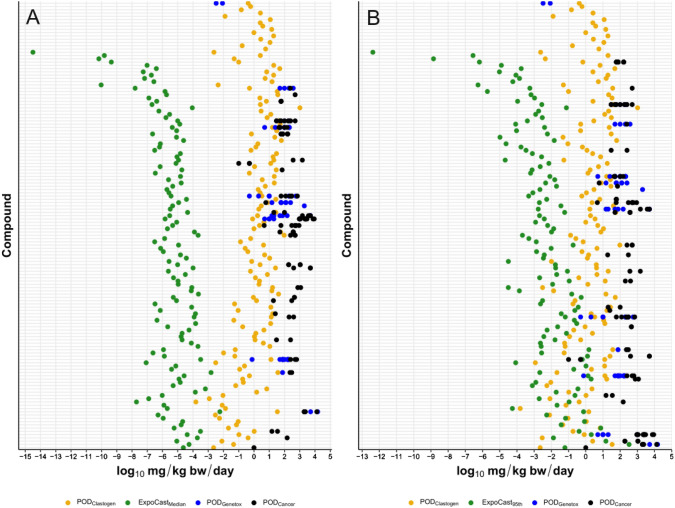
Bioactivity Exposure Ratios (BERs) for compounds classified as clastogens based on the decision tree (yellow). **A** ExpoCast median exposure estimates (green), and **B** ExpoCast 95th percentile exposure estimates (green). Traditional genetic toxicology POD (blue) and cancer POD (black) from ToxValDB are plotted for comparison. The full list of chemicals and their respective POD values are in Supplemental File 8 (color figure online)

**Fig. 6:**
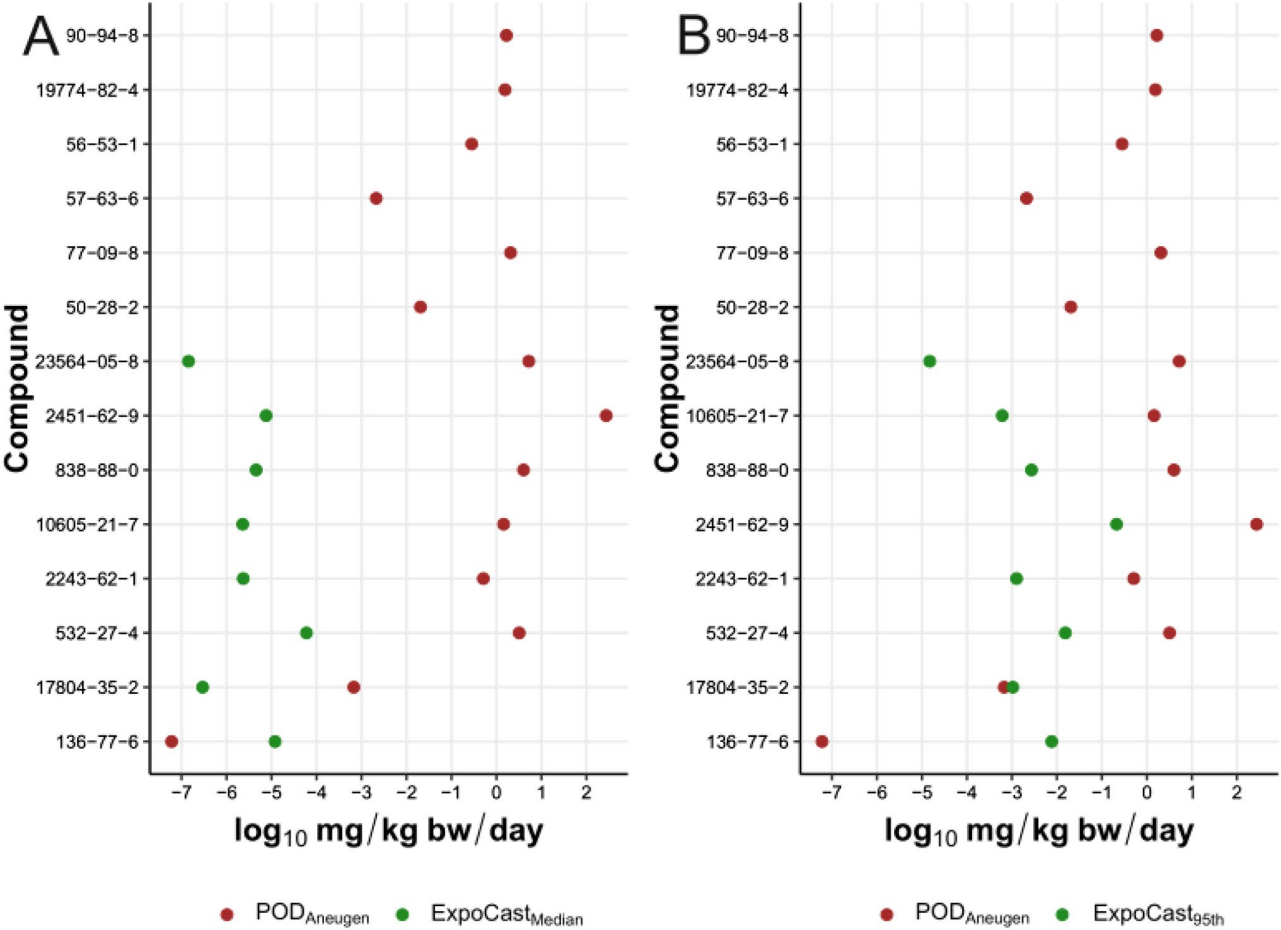
Bioactivity Exposure Ratios (BERs) for compounds classified as aneugens based on the Bryce et al. (2011) approach (red). **A** ExpoCast median exposure estimates (green); **B** ExpoCast 95th percentile exposure estimates (green) (color figure online)

Available exposure estimates allowed for the derivation of BERs for 122 clastogens (Fig. 5) and 8 aneugens (Fig. 6; details in Supplemental File 1). Using the ExpoCast median exposure estimate, there was one clastogenic compound with a log_10_BER < 2 (dinoseb, log_10_BER = 1.98), two compounds with a log_10_BER between 2 and 3 (azobenzene, log_10_BER = 2.92; 1,2-diphenylhydrazine, log_10_BER = 2.96), and the remaining 119 compounds had a log_10_BER > 3 (range 3.33–11.87). Using the ExpoCast 95th percentile exposure estimates, there were seven compounds with negative log_10_BERs, where exposure estimates were above AEDs based on MN data. Specifically, dinoseb, 1,2-propylene glycol, caprolactam, 4-nitroaniline, FD&C yellow 5, 1,2-dimethyl-3-nitrobenzene, and di(2-ethylhexyl) adipate had log_10_BERs ranging from − 1.44 to − 0.04. There were 43 chemicals where the log_10_BER was between 0 and 2 (range 0.09–1.99), 23 chemicals with log_10_BER between 2 and 3 (range 2.05–2.97), and 49 chemicals with log_10_BER > 3 (range 3.00–9.75).

When the ExpoCast median exposure estimates were used to evaluate chemicals classified as aneugenic, there was one compound with a negative log_10_BER. Specifically, 4-hexylresorcinol had a log_10_BER of − 2.30. This was driven by the extremely low BMC (i.e., 1 × 10^–6^ µM without S9) rather than by the *C*_*ss*_ or exposure estimate. The remaining 7 compounds all had a log_10_BER > 3 (range 3.36–7.56). When the derivation of BERs for aneugenic compounds was based on 95th percentile ExpoCast exposure estimate, there were two compounds with negative log_10_BERs. These were 4-hexylresorcinol (− 5.11) and benomyl (− 0.19). There were no compounds with a log_10_BER between 0 and 2. There were two compounds with a log_10_BER between 2 and 3: 2-chloroacetophenone (2.32) and 1,5-naphthalenediamine (2.60). The remaining four compounds had a log_10_BER > 3 (triglycidyl isocyanurate, 3.11; 4,4-methylenebis(2-methylaniline), 3.16; carbendazim, 3.37; thiophanate-methyl, 5.55).

## Discussion

Chemical genotoxicity assessment typically requires a combination of rigorous in vitro and in vivo studies, which can be costly and time-consuming. In this study, we paired in vitro and in silico methodologies to classify and assess the genotoxicity of 292 chemicals using a modified experimental design for the high-throughput MNvit assay. Our approach consisted of four parts: (1) a decision tree to classify the chemicals based on MNvit data as positive, negative or inconclusive, (2) BMC analyses to identify a POD, (3) a toxicokinetic pipeline for IVIVE to produce AEDs for comparison to existing in vitro and traditional toxicity PODs; and (4) derivation of BERs through comparison of in vitro genotoxicity PODs with human exposure estimates for prioritization. We demonstrate how our approach can be used for hazard identification through identifying chemicals inducing significant concentration-responses, as well as for deriving PODs for each chemical. In addition, application of IVIVE enables conversion of the in vitro POD values to AEDs for comparison to in vivo responses and human exposure levels. Overall, the work describes an approach that can be used for weight-of-evidence evaluation as well as for potency comparisons and chemical prioritization.

In a typical analysis of MNvit dataset, a positive genotoxicity assessment requires a statistically significant increase in MN frequency in at least one concentration over solvent-matched and historical controls (OECD 2016). In our datasets, there were 19 concentrations tested per chemical, enabling a robust quantitative analysis to increase the precision of hazard assessment and POD derivation. The highest tested concentration was 200 µM, which is over the cytotoxicity threshold for most chemicals. With a few exceptions, this top concentration is also well above expected exposure levels for environmental chemicals. This design allowed us to bypass the typical pilot studies needed to establish test concentrations. A typical MN analysis might include 12–18 sample in total to investigate 3–5 concentrations, plus solvent controls. Instead, despite the lack of concentration-specific replicates, the experimental design herein includes more samples to more precisely define the concentration–response relationship. To ensure that the approach is robust, we designed a decision tree-based assessment approach that includes the use of cytotoxicity assessment, filtering to identify increases above batch solvent control, trend analyses, filtering to ensure at least one response is > 2 × the solvent control, and concentration–response modeling to fit curves and identify BMCs for each chemical. The decision tree began with assessing the response of each concentration against the 95^th^ percentile of batch-specific controls. We required at least two consecutive concentrations below or at the cytotoxicity threshold that were also over the 95th percentile of the controls prior to concentration–response analyses to ensure that the damage observed was not the result of cytotoxicity. In addition to requiring a concentration–response trend, filtering to ensure that the BMC was less than or equal to the highest concentration applied (i.e., below the cytotoxic threshold) further eliminated chemicals not meeting minimal positive responses within the concentration range tested. For most chemicals, the number of remaining concentrations applied in the BMC analysis following this filtering for cytotoxicity was more than ten, which provided robust BMC values.

The MNvit assay operates through detection of DNA fragments or whole chromosomes that manifest in the cytoplasm as micronucleus events after cell division. Thus, an increase in %MN can be the result of clastogenic or aneugenic (hypodiploidy) mechanisms. We attempted to assess both MN and hypodiploidy % with the proposed decision-tree approach. The decision tree classified 157 (53.7%) of the chemicals as MN positive, 25 (8.6%) negatives and 110 (37.7%) inconclusive. The optional two-fold filter reclassified 16 MN positive chemicals to negative and 26 to inconclusive. The same approach classified 209 (71.6%) chemicals as aneugenic positives, 27 (9.2%) negatives and 56 (19.2%) inconclusive. Conversely, the approach proposed by Bryce et al. (2011) classified 15 (5.1%) of these chemicals as positives and 277 (94.9%) as negatives. Since the number of positive calls using our initial approach was much higher than expected based on the number of known aneugens, we have more confidence in the Bryce et al. (2011) approach, indicating 5% of the chemicals are aneugens. Indeed, by manually examining the 15 classified aneugens, we found that seven and three chemicals were true or potential aneugens based on published data (Supplemental File 3) and there were no conflicting data suggesting otherwise for the others. Thus, assuming that these 15 chemicals induced MN through aneugenic mechanisms, leads us to conclude that the remaining 142 chemicals (of the 157 yielding positive calls) are potential clastogens.

Although the results from the proposed classification approaches can help prioritizing positive clastogens and aneugens for further investigation, the number of chemicals is substantial for comprehensive follow-up testing. Thus, we applied IVIVE to predict the AEDs of these clastogenic and aneugenic chemicals (i.e., POD_Clastogen_ and POD_Aneugen_, respectively), and compared them to exposure estimates to enable prioritization based on potential risk. To establish confidence in the AEDs, we compared them with known genetic toxicity and cancer PODs. The closer a POD_Clastogen_ or POD_Aneugen_ for a compound was to its known traditional POD, the less conservative, or more aligned, the IVIVE derived POD was. For compounds where the AED was not protective of human health, follow-up investigations using higher tier physiologically based toxicokinetics models or other genetox assays may be required to understand the discrepancies with in vivo data.

Comparisons of POD_Clastogen_ with traditional PODs, based on in vivo genotoxicity or cancer studies, revealed that the POD_Clastogen_ was protective (i.e., lower) for most compounds. Specifically, the POD_Clastogen_ was lower than the POD_Cancer_ for 83.9% of compounds, and on average was lower by approximately 14-fold. Similarly, the POD_Clastogen_ was lower than the POD_Genetox_ for 72.7% of compounds, on average by approximately sixfold. Previous work comparing PODs based on in vitro bioactivity data from the ToxCast battery, found that the bioactivity PODs were lower than traditional PODs from repeat-dose studies for 89% of chemicals by > 100-fold (Paul Friedman et al. 2020). Thus, MNvit is not as sensitive as the ToxCast test battery, but it is impressive this single assay provided protective PODs in a majority of cases.

Acrylamide was the only compound where the POD_Clastogen_ was two orders of magnitude higher than the POD_Cancer_. The genotoxicity of acrylamide has been extensively studied, and it is well-established that acrylamide is a clastogenic compound at relatively high doses (European Food Safety Authority 2015). In addition, the active metabolite of acrylamide, glycidamide, is a strong mutagen as well as a clastogen. The POD_Cancer_ values used in the comparison with the POD_Clastogen_ were based on a two year cancer study in rats where acrylamide was administered through drinking water (Johnson et al. 1986). The lowest POD_Cancer_ was a no-observed-adverse-effect level (NOAEL) of 0.1 mg/kg for mesothelioma of the testes tunica albuginea. The other two POD_Cancer_ values were based on NOAELs of 0.5 mg/kg for mammary gland tumors or thyroid gland tumors. These tissues are hormone-sensitive, and it has been postulated that there are alternative modes of action than genotoxicity driving the formation of these tumors, and that the modes of action for scrotal mesothelioma tumor response may not be relevant to humans (Haber et al. 2009; Hogervorst et al. 2010). In addition, it has been shown that carcinogenicity occurs at lower doses than genotoxicity in both rat and mouse models, supporting alternative modes of action (Chepelev et al. 2017; Hobbs et al. 2016). Indeed, gene expression analysis of rat testis following acrylamide exposure found that the main transcriptional alterations were associated with cytoskeletal proteins and calcium signaling, with no strong evidence for the induction of genotoxicity (Recio et al. 2017). Thus, a more reliable evaluation of the POD_Clastogen_ would be to compare it with PODs derived from in vivo MN studies. Examining rodent studies measuring in vivo MN induced by acrylamide (Rothfuss et al. 2010; Yener 2013) showed that the lowest POD_Genetox_ is 2.0 mg/kg bw/day based on a lowest-observed-adverse-effect level (LOAEL) in polychromatic erythrocytes of male rats (Yener 2013) and a NOAEL in normochromatic erythrocytes of male mice (Zeiger et al. 2009). Moreover, Hobbs et al. (2016) found that B6C3F1 mice exposed to 6 mg/kg bw/day of acrylamide had increased frequencies of micronucleated reticulocytes and normochromatic erythrocytes. However, the same response was not identified in F344 rats at doses as high as 12 mg/kg bw/day, which is lower than the POD_Clastogen_ of 12.6 mg/kg bw/day. Hence, the POD_Clastogen_ more closely aligns with the POD_Genetox_, with the POD_Clastogen_ being 2- to 6-fold higher than the POD_Genetox_ for positive results.

Folic acid was the only compound with a POD_Clastogen_ more than 100-fold higher than the POD_Genetox_. However, the POD_Genetox_ was based on folate deficiency and this is not addressed by our study. Manufactured folic acid is a dietary supplement that is used in food fortification and is converted in the body to folate, a water-soluble naturally occurring B vitamin. It is recommended that pregnant women are supplemented with an extra 0.4 mg of synthetic folic acid per day to reduce the chance of neural tube defects in their offspring, and the tolerable daily intake of folic acid is 1 mg/day (Institute of Medicine (US) Standing Committee on the Scientific Evaluation of Dietary Reference Intakes and its Panel on Folate, Other B Vitamins, and Choline 1998). There is strong evidence that folic acid deficiency is associated with increased cancer risk and elevated rates of mutations and micronuclei (LeBlanc et al. 2018; National Toxicology Program 2015), and that the response may be tissue-dependent (Diaz et al. 2021). Indeed, the folic acid POD_Genetox_ of 3 × 10^–3^ mg/kg bw/day (i.e., intake of 0.195 mg/day in a 60 kg adult) used in the comparison with POD_Clastogen_ was based on the baseline folic acid level in postmenopausal women, and folic acid levels that were one third below this level were associated with increased MN in lymphocytes and exfoliated buccal cells (i.e., representing levels of folic acid deficiency) (Titenko-Holland et al. 1998). Thus, the POD_Clastogen_ should not be viewed as being non-protective of POD_Genetox_, but instead the POD_Clastogen_ raises questions about the relationship between excessively high levels of folic acid and micronuclei induction, which require further study. There is evidence that relatively high levels of folic acid may be weakly associated with colorectal cancer under select circumstances and through an unknown mechanism (Kim 2006, 2018). A recent study demonstrated that mice supplemented with a folic acid dose of 8 mg/day (i.e., approximately a diet of 1.6 mg/day in a human) had elevated mutation frequencies in colon but not bone marrow (Diaz et al. 2021). The POD_Clastogen_ derived in this study was 0.4 mg/kg bw/day (24 mg/day in 60 kg adult; 30 mg/day in 75 kg adult), and far exceeds the established tolerable daily intake for folic acid. Consequently, any potential risk associated with clastogenicity can be considered negligible relative to the benefits of supplementary folic acid intake, and the guidance for folic acid supplementation should be followed (Koren 2011).

We explored using MNvit assay to identify aneugenic compounds. Using the decision-tree approach, an unexpectedly high number of aneugens totaling 209 chemicals were identified. In contrast, the Bryce et al. (2011) approach classified 15 chemicals as aneugens, seven of which are known aneugens and three are likely aneugens. Specifically, three benzimidazole compounds, benomyl, carbendazim, and methyl thiophanate (parent compound of carbendazim), are well-established aneugens (Barale et al. 1993; Van Hummelen et al. 1995), and were classified accordingly in this study. The naturally occurring steroid hormone 17*β*-estradiol and synthetic analogs, 17*α*-ethinylestradiol and diethylstilbestrol, are also established aneugens (Brown et al. 2008; Hernández et al. 2013; Parry et al. 2002; Rosefort et al. 2004) and our MNvit results support these findings. The other chemicals with elevated hypodiploidy levels have been previously shown to be aneugenic, such as Michler’s ketone (Lafi et al. 1986) and phenolphthalein (Armstrong et al. 2000), or test positive for other chromosomal aberrations, such as triglycidyl isocyanurate (Willcocks et al. 1998). These comparisons support the utility of the Bryce et al. (2011) approach in identifying aneugenic compounds, and thus, chemicals classified as aneugenic using this approach should be considered as priorities for further scoping and risk evaluation.

Based on the BER analysis, 4-hexylresorcinol, an antiseptic and food additive, has a high potential for aneugenic concern due to its very low POD_Aneugen_ and BER. The POD_Aneugen_ for 4-hexylresorcinol was two and five orders of magnitude lower than the ExpoCast median and 95^th^ percentile estimates, respectively. The low POD_Aneugen_ of 6 × 10^–8^ mg/kg bw/day results from the low BMC (1 × 10^–6^ μM) as opposed to the HTTK-modeled *C*_*ss*_ used for POD derivation. The *C*_*ss*_ is not expected to be high as in vivo studies have shown that 4-hexylresorcinol is rapidly excreted in urine and feces (Walker 2005). Interestingly, previous investigations of 4-hexylresorcinol using the MN assay found that it was negative in vitro and positive in vivo (Soeteman-Hernández et al. 2015, 2016). In our study, 4-hexylresorcinol was considered negative for clastogenicity classification with and without S9. Likewise, resorcinol is the precursor for 4-hexylresorcinol, which has been shown to be negative for MN in vivo (Natarajan and Obe 1986). Resorcinol is an ECVAM group 3 chemical, meaning it yields positive in vitro results at high concentrations, likely as a result of high levels of cytotoxicity (Kirkland et al. 2008, 2016). In contrast, there is evidence that 5-pentylresorcinol, another structural analog of 4-hexylresorcinol, may disrupt microtubule and spindle formation through a decrease in DNA synthesis and indirect induction of abnormal anaphase configurations (National Toxicology Program 1988), indicating that it is a potential aneugen. In the EFSA re-evaluation of 4-hexylresorcinol as a food additive, the panel concluded that there was no safety concern of genotoxicity or carcinogenicity for 4-hexylresorcinol based on its current use levels (European Food Safety Authority 2014). However, the panel noted that there were no reproductive and developmental toxicity studies, and that a one-generation study would be required before permitting an increase in usage level. Based on the findings of this study, an in vivo assessment of aneugenicity should also be considered for any assessments of 4-hexylresorcinol.

There are several reasons why the PODs derived using our computational approach may differ from traditional PODs. First, the PODs were derived using a BMC of one standard deviation (30% for micronucleus and 60% for hypodiploidy), whereas the traditional PODs are often based on NOAELs confined by study design. Second, the IVIVE model was limited to the toxicokinetic parameters of the parent compound. This is a suitable approach when S9 activation is not required, as was the case for the majority of clastogens (118/157) and aneugens (12/15). However, for compounds where the metabolite is active and drives genotoxicity, there are several assumptions that need to be made for IVIVE (Lutz et al. 2010). Specifically, we made conservative assumptions that the formation clearance rate of the active metabolite was approximately the same as the elimination clearance rate, the active metabolite is the main metabolite from biotransformation of the parent and approaches the same concentration in the plasma as the parent, and the metabolite is available to the systemic circulation. In general, this approach yielded protective PODs from the MNvit data; however, more focused research on predicting the in vivo disposition of metabolites is required to improve the approach. Third, the PODs were derived using the nominal (assumed) concentration in the medium as opposed to the true intracellular concentration, which can vary by orders of magnitude for certain chemical classes (Armitage et al. 2014). Overall, further refinements to the computational approach will serve to enhance the utility of this novel quantitative in vitro genetic toxicology framework for risk assessment applications.

Another important consideration is that there are several modes of action underlying genotoxicity and carcinogenicity, and one assay alone is often not sufficient for hazard characterization of genotoxicants. The development of a validated panel of complementary higher-throughput and higher-content in vitro assays could greatly enhance the mechanistic information and concomitantly improve genotoxicity assessment. Several higher-throughput assays have recently been developed for assessing genotoxicity and most of these assays are functionally related to well-established assays with existing test guidelines. Another complementary assay for detecting gene mutations is the in vitro MutaMouse assay that detects mutations in the *lacZ* reporter gene in FE1 cells (Maertens et al. 2017; White et al. 2003). Furthermore, there are novel assays that detect biomarkers of DNA damage response using flow cytometry (MultiFlow) (Bemis and Heflich 2019; Bryce et al. 2018) or using reporter cell lines (ToxTracker) (Hendriks et al. 2012). Higher content assays, such as the TGx-DDI biomarker (Li et al. 2015, 2017) provide a robust classification system for identifying DNA-damaging agents, and aid in better understanding a chemical’s mode of action. In addition, the choice of cell line and the efficacy of the S9 metabolic activation, and other factors, may influence the PODs derived from the in vitro assays. Thus, application of a combination of these in vitro assays in a variety of cell lines, with an integrated computational approach, could serve to identify compounds with the highest potential for genotoxic concern as priorities for further work.

In summary, we propose a framework for the use of a modified test paradigm of MNvit datasets for genotoxic hazard identification, potency analysis and compound prioritization. This study is aligned with recent efforts to advance the use of genotoxicity data, which has traditionally been used exclusively for hazard identification, toward quantitative evaluation and application in risk assessment to support regulatory decision making (Heflich et al. 2020; White et al. 2020). We applied IVIVE to extrapolate the BMC values of the positive clastogens and aneugens to derive AEDs, enabling comparison with traditional genotoxicity and cancer PODs. The majority of the determined AEDs were protective relative to POD_Genetox_ and POD_Cancer_. Overall, our proposed approach enables prioritization of compounds for further evaluation on the basis of BERs and the use of our decision-tree pipeline supports weight-of-evidence based decision making in the context of effective risk assessment.

## Supplementary Information

Below is the link to the electronic supplementary material.

Supplementary file1 Supplemental File 1. IVIVE analysis output of MN positive and Hypodiploidy positive chemicals. (XLSX 81 KB)

Supplementary file2 Supplemental File 2. Datasets pooled from flow cytometry MNvit test. Includes cytoxicity, MN and Hypodiploidy counts, percentages, and fold changes. (XLSX 925 KB)

Supplementary file3 Supplemental File 3. Detailed assessment results for MN and Hypodiploidy based on decision tree (clastogen and aneugen) and Bryce et al. 2011 (aneugen). (XLSX 591 KB)

Supplementary file4 Supplemental File 4. Manual assessment of 15 aneugens classified using methods described in Bryce et al. 2011. (XLSX 13 KB)

Supplementary file5 Supplemental File 5. Ranked BMC confidence interval plot of MN positive chemicals based on the decision-tree approach. (PDF 13 KB)

Supplementary file6 Supplemental File 6. Ranked BMC confidence interval plot of Hypodiploidy positive chemicals based on the decision-tree approach. (PDF 14 KB)

Supplementary file7 Supplemental File 7. Ranked BMC confidence interval plot of Hypodiploidy positive chemicals based on the approach described in Bryce et al. 2011. (PDF 5 KB)

Supplementary file8 Supplemental File 8. PODclastogen vs traditional genetox and cancer PODs. (XLSX 16 KB)

## Declarations

### Disclaimer

This article has been reviewed by the U.S. Environmental Protection Agency, Office of Research and Development and approved for publication. Approval does not signify that the contents reflect the views of the Agency, nor does mention of trade names or commercial products constitute endorsement or recommendation for use.
