# Supplementary figures and images for: Comprehensive interpretation of in vitro micronucleus test results for 292 chemicals: from hazard identification to risk assessment application
Source: Arch Toxicol. 2022 Apr 21;96(7):2067–85. doi: 10.1007/s00204-022-03286-2 (PMC9151546; doi:10.1007/s00204-022-03286-2)

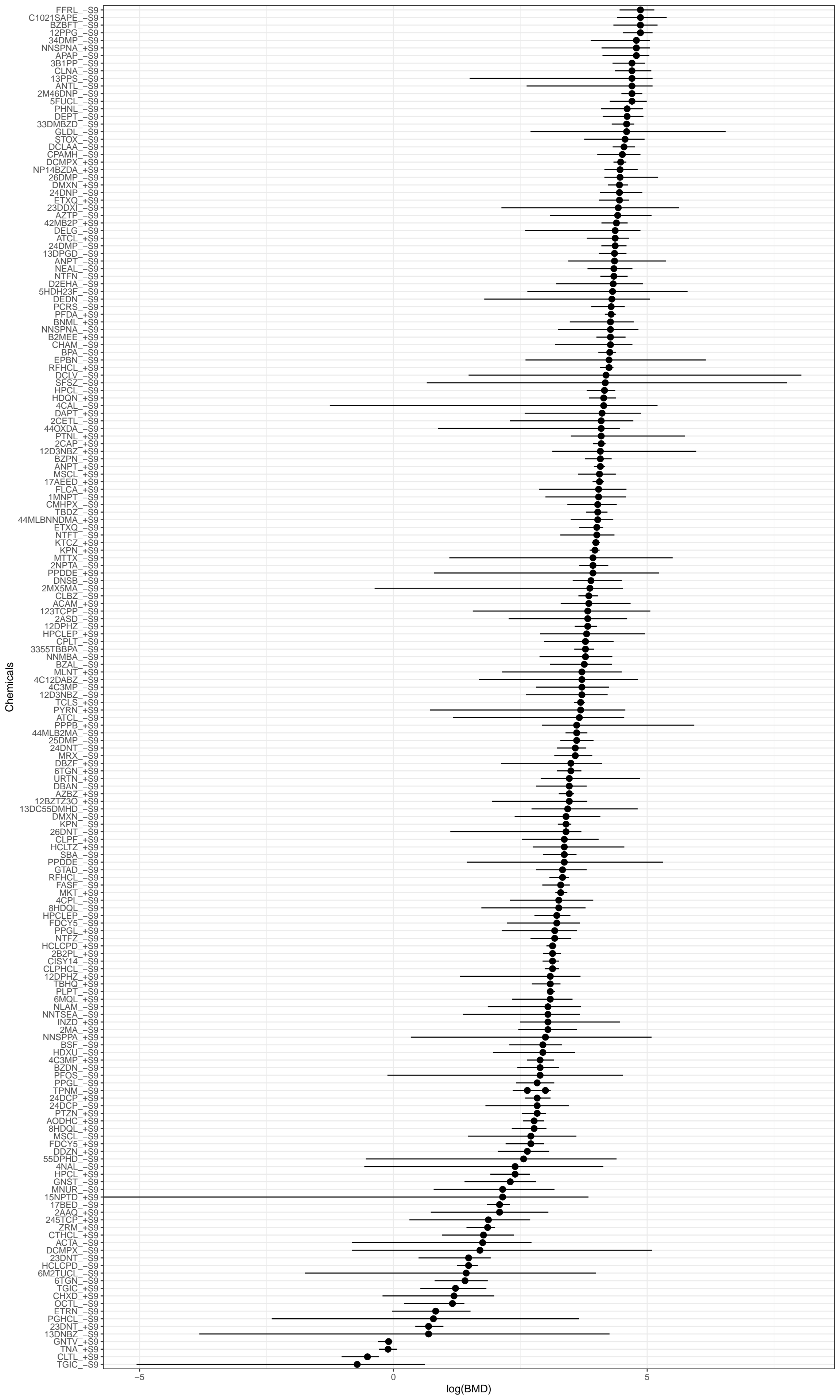

Supplement: Supplementary file 5 — Supplementary file5 Supplemental File 5. Ranked BMC confidence interval plot of MN positive chemicals based on the decision-tree approach. (PDF 13 KB) [file 204_2022_3286_MOESM5_ESM.pdf]

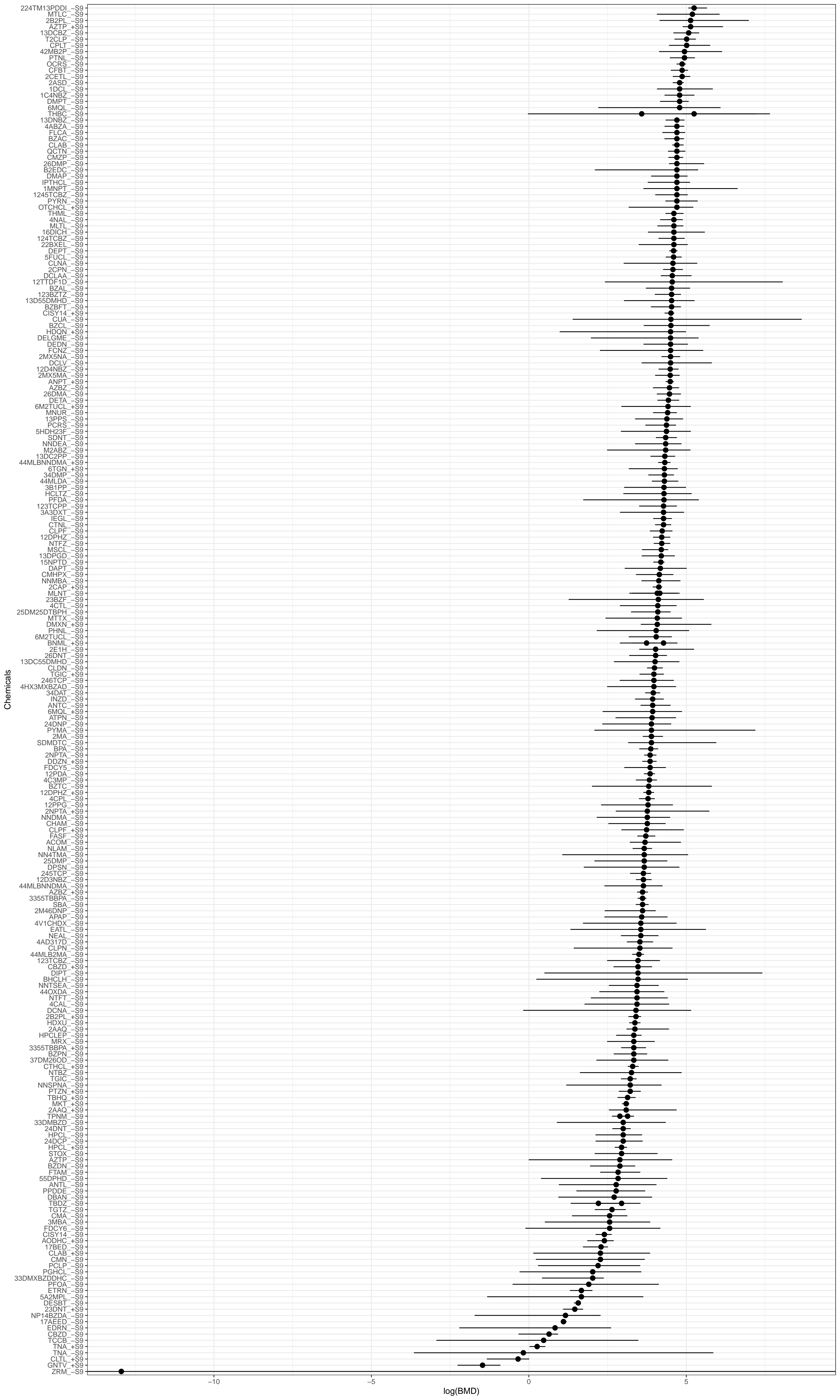

Supplement: Supplementary file 6 — Supplementary file6 Supplemental File 6. Ranked BMC confidence interval plot of Hypodiploidy positive chemicals based on the decision-tree approach. (PDF 14 KB) [file 204_2022_3286_MOESM6_ESM.pdf]

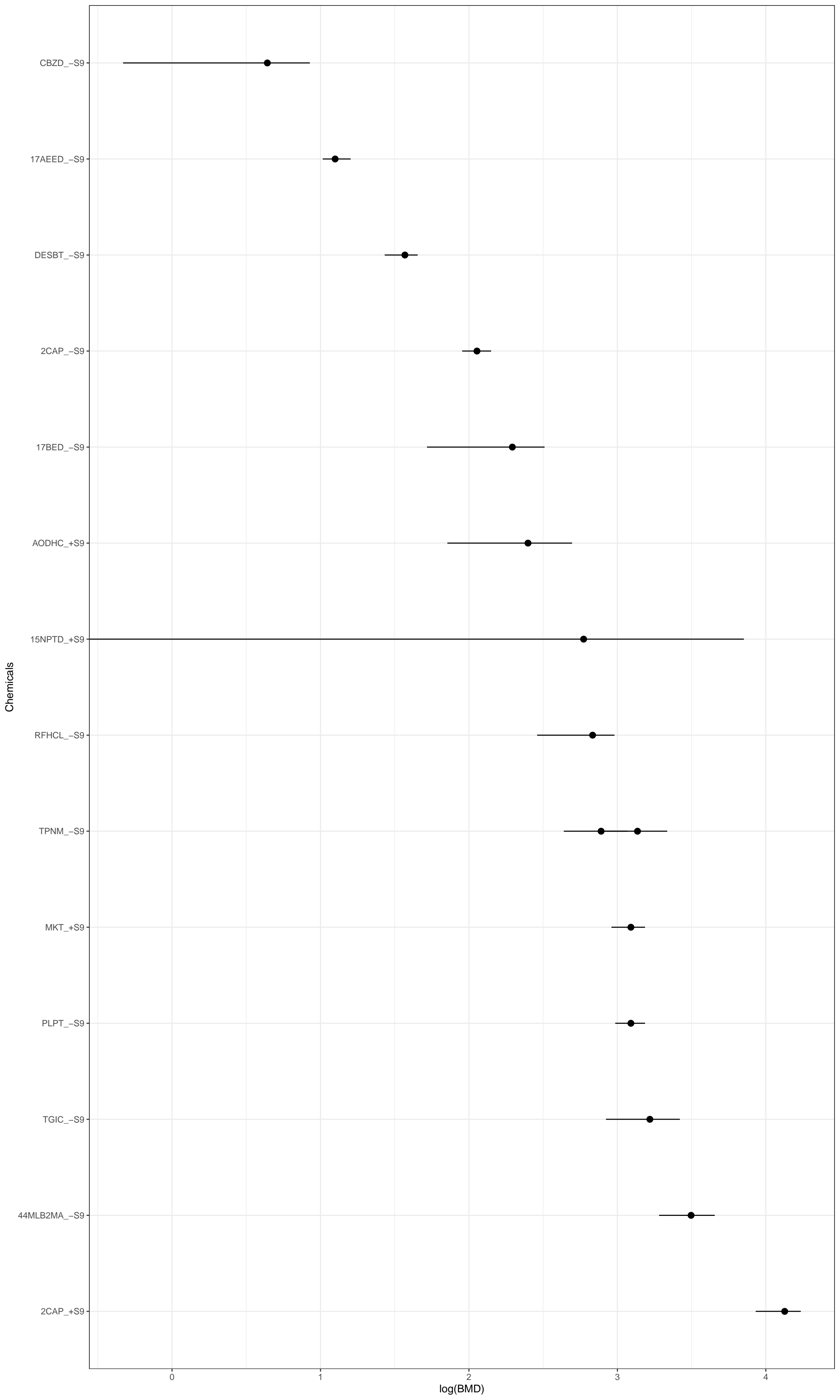

Supplement: Supplementary file 7 — Supplementary file7 Supplemental File 7. Ranked BMC confidence interval plot of Hypodiploidy positive chemicals based on the approach described in Bryce et al. 2011. (PDF 5 KB) [file 204_2022_3286_MOESM7_ESM.pdf]
